# Lymphoma in Meckel cave mimicking trigeminal schwannoma – analysis of misdiagnosis causes and insights into imaging: Case report

**DOI:** 10.1097/MD.0000000000047137

**Published:** 2026-01-09

**Authors:** Tong Zhang, Xiaowen Liu, Qinghua Zhang, Qiang Li, Chengxin Yan

**Affiliations:** a Department of Medical Imaging, The Second Affiliated Hospital of Shandong First Medical University, Taian, Shandong, China.

**Keywords:** central nervous system lymphoma, Meckel cave, trigeminal neuralgia, trigeminal schwannoma

## Abstract

**Rationale:**

Central nervous system lymphoma typically arises in the deep cerebral regions, while occurrence in the Meckel cave at the skull base is exceedingly rare, with fewer than 1 hundred cases reported globally. Lesions in this area are often misdiagnosed as trigeminal schwannomas or meningiomas. This report aims to enhance clinical recognition of this rare entity by presenting a meticulously analyzed case that was initially misdiagnosed as a trigeminal schwannoma due to overlapping radiological features. Through detailed clinicopathological correlation, we seek to identify key diagnostic indicators that may facilitate earlier accurate diagnosis and prevent unnecessary surgical interventions.

**Patient concerns:**

A 60-year-old female presented with a 1-month history of paroxysmal electric shock-like pain in the right ala nasi, cheek, and upper lip. Symptoms occurred multiple times daily without identifiable triggers and were not accompanied by other neurological deficits. The patient had no history of immunodeficiency-related conditions.

**Diagnoses:**

Imaging revealed a space-occupying lesion in the right Meckel cave, demonstrating a “dumbbell-shaped” transcompartmental growth pattern along the trigeminal nerve pathway. The lesion was misdiagnosed preoperatively as a trigeminal schwannoma. Postoperative pathology confirmed diffuse large B-cell lymphoma, with immunohistochemistry indicating a germinal center subtype.

**Interventions:**

The patient underwent surgical resection to relieve nerve compression. Due to the preoperative misdiagnosis, cerebrospinal fluid cytology and comprehensive systemic staging were not performed.

**Outcomes:**

Facial pain resolved postoperatively. However, the patient continued to experience residual facial numbness postoperatively. Given the confirmed lymphoma diagnosis, the patient is scheduled to receive methotrexate-based combination chemotherapy following the postoperative recovery period.

**Lessons:**

The “perineural growth” pattern and “dumbbell” morphology of this Meckel cave lymphoma were highly deceptive and directly led to misdiagnosis. Lymphoma should be critically included in the differential diagnosis for Meckel cave lesions presenting with rapid progression (<6 months), schwannoma-like imaging features, and absence of prominent T2 hyperintensity. Preoperative cerebrospinal fluid analysis or targeted biopsy can prevent unnecessary surgical interventions and ensure timely administration of standardized chemotherapy.

## 1. Introduction

Central nervous system lymphoma (CNSL) predominantly arises in the deep cerebral white matter.^[1]^ In contrast, CNSL originating from the skull base or cranial nerves is exceptionally rare. The Meckel cave is a critical anatomical structure in the central skull base. It houses the trigeminal ganglion, cerebrospinal fluid, and small vasculature, while connecting to adjacent facial compartments such as the infratemporal fossa and pterygopalatine fossa through natural foramina. This unique architecture enables tumors to develop in a “dumbbell-shaped” configuration spanning multiple cranial compartments. The most prevalent tumors in this region are trigeminal schwannomas and meningiomas.^[2]^ Lymphoma at this site is exceptionally rare, with only scant cases documented globally. This article presents a case of diffuse large B-cell lymphoma (DLBCL) originating in Meckel cave. The lesion exhibited imaging characteristics closely mimicking trigeminal schwannoma – demonstrating perineural extension along the trigeminal pathway and a classic transcompartmental “dumbbell” morphology – which culminated in preoperative misdiagnosis.

## 2. Case report

### 2.1. Clinical presentation

A 60-year-old female patient presented with a 1-month history of paroxysmal electric shock-like pain affecting the right ala nasi, cheek, and upper lip. Symptoms occurred multiple times daily without identifiable triggers. No concomitant headache, nausea, gait instability, or other cranial nerve deficits were reported. Medical history revealed no autoimmune disorders or immunosuppressive therapy. Physical examination, complete blood count, hepatic/renal function, and serum electrolytes were unremarkable.

### 2.2. Computed tomography and magnetic resonance imaging findings (non-contrast + contrast-enhanced)

**Figure 1.**
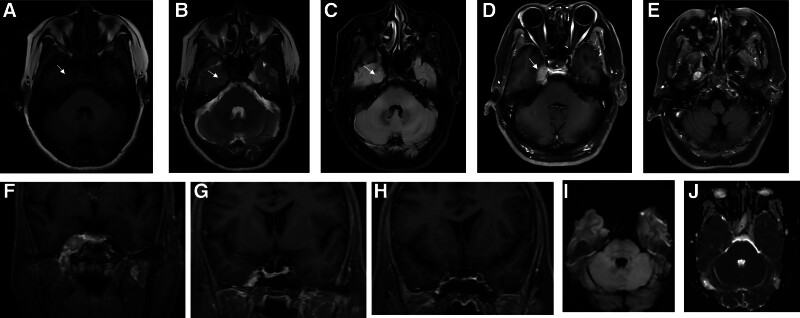
(A) T1WI, (B) T2WI, and (C) T2-FLAIR sequences demonstrate the lesion (arrow) exhibiting isointensity to brain parenchyma. (D and F) Axial and coronal T1-weighted contrast-enhanced MRI show that the parasellar mass-like lesion had obvious heterogeneous enhancement, with unclear boundary from the trigeminal nerve, and invaded the cerebellopontine angle area through the “Meckel cave.” (E) Axial T1-weighted enhanced MRI shows tumor extension through the foramen ovale into the infratemporal fossa. (G and H) Coronal T1-weighted enhanced MRI confirms involvement of the lateral wall of the right cavernous sinus. (I and J) DWI and ADC demonstrate mild diffusion restriction, evidenced by partially hyperintense DWI signals and correspondingly hypointense ADC values within the lesion. ADC = apparent diffusion coefficient, DWI = diffusion-weighted imaging, MRI = magnetic resonance imaging.

**Figure 2.**
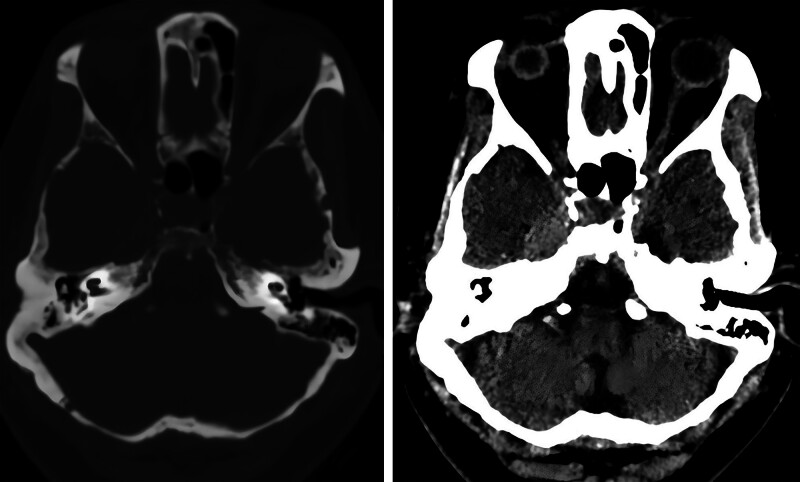
CT imaging shows no evidence of calcification, hemorrhage, necrosis, or significant bony hyperostosis/destruction within detectable limits. CT = computed tomography.

MRI (Fig. 1) shows a right paranasal mass with isointense signal on T1-weighted and T2-weighted images, measuring approximately 15 mm × 12 mm. The lesion extends to the lateral wall of the cavernous sinus but does not encircle the internal carotid artery, with unclear boundaries from the right trigeminal nerve. It extends posteriorly into the posterior cranial fossa via the Meckel cave and downward into the infratemporal fossa via the foramen ovale (between the palatopharyngeus muscle and the pterygoid muscle). Contrast-enhanced scans revealed marked heterogeneous enhancement. CT (Fig. 2) reveals an irregular, slightly hyperdense shadow in the right parasellar region with indistinct borders. No obvious bone disruption or destruction is seen in the parasellar bone. Preliminary imaging diagnosis: high likelihood of trigeminal nerve schwannoma.

### 2.3. Surgical and pathological findings

The patient underwent resection of the skull base lesion. Intraoperatively, a firm, well-encapsulated tumor was adherent to the right trigeminal nerve in the parasellar region, with moderate vascularity. The mass protruded into the foramen ovale and extended posteriorly through Meckel cave into the posterior fossa.

### 2.4. Histopathological analysis

**Figure 3.**
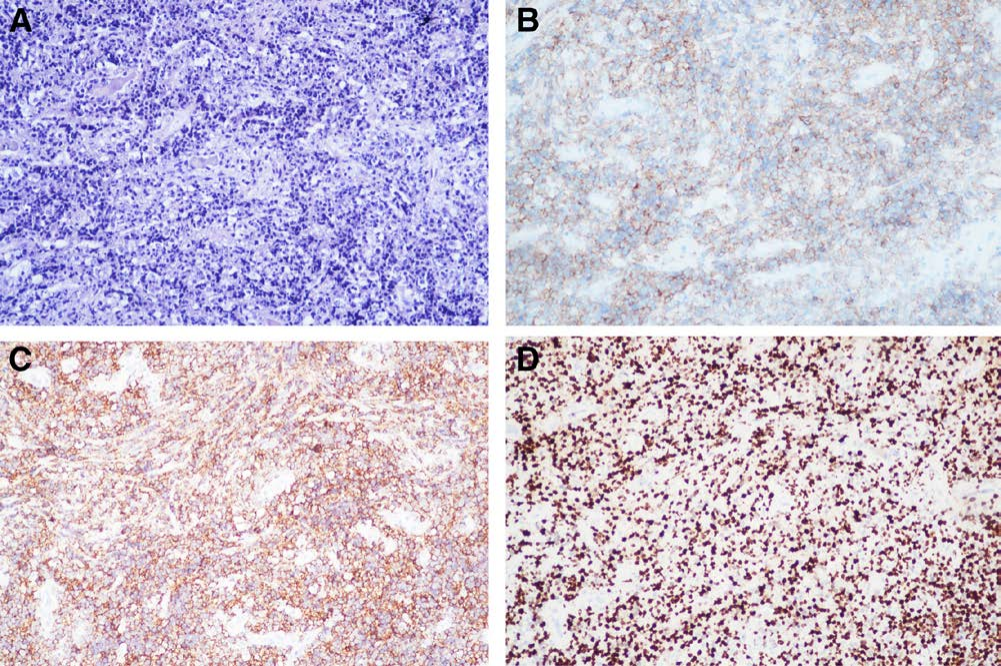
(A) Hematoxylin and eosin staining (×200) demonstrates diffuse infiltrative growth of medium-sized lymphoid cells with focal necrosis and readily identifiable mitotic figures. (B–D) Immunohistochemical staining (×200) reveals CD19(+), CD20(+) and Ki-67 (80%+).

Microscopy (Fig. 3): Diffuse infiltrative growth of medium-sized lymphoid cells, focal necrosis, and frequent mitotic figures. Immunohistochemistry (Fig. 3): Positive: CD20, CD19, CD10, Bcl-6 (80%), Bcl-2 (90%), MUM-1, C-Myc (20%), CD5 (partial); Negative: S-100, SOX-10, EMA, CKpanWe, ALK, CyclinD1, TdT; Proliferation/Apoptosis: Ki-67 (80% in hotspots), P53 (wild-type); T-cell Marker: CD3 (background T-cells+); Diagnosis: Aggressive B-cell lymphoma, consistent with diffuse large B-cell lymphoma (DLBCL), germinal center B-cell(GCB).^[3]^

### 2.5. Clinical course

Postoperative alleviation of trigeminal neuralgia was observed, with a multimodal therapeutic regimen planned following the convalescence period. However, the patient continued to experience residual facial numbness postoperatively.

## 3. Discussion

Lymphoma involving Meckel cave represents an atypical localization of CNSL. Its clinical and imaging features closely mimic common pathologies such as schwannoma or meningioma, posing diagnostic challenges. In the present case, the patient presented with unilateral paroxysmal neuropathic facial pain, clinically indistinguishable from classical trigeminal neuralgia. Notably, approximately 90% of trigeminal neuralgia cases arise from vascular compression and demyelination at the root entry zone of the pontine trigeminal nerve, while only 10% are attributed to neoplastic etiologies.^[4,5]^

Meckel cave lymphoma is exceptionally rare, with some cases originating from adjacent regions (e.g., orbit or cavernous sinus).^[6,7]^ Importantly, such skull base lesions are frequently misdiagnosed as meningiomas or schwannomas in the literature.^[2]^ Previously reported Meckel cave lymphomas presenting with trigeminal neuralgia-like symptoms uniformly involved all trigeminal branches and exhibited progressive neurological deficits.^[8]^ Previously documented cases of Meckel cave lymphoma uniformly demonstrate diffuse involvement of all trigeminal nerve divisions (V1-V3) with progressive sensory deficits or motor impairments. In stark contrast, the present case exhibited isolated mandibular nerve (V3) involvement, manifesting as paroxysmal electric shock-like pain in the right cheek and mental region without concomitant neurological deficits. Kinoshita et al described a similar case extending through the foramen ovale into the infratemporal fossa.^[8]^ Due to rapidly progressive neurological deficits unexplained by imaging, diagnosis was confirmed via cerebrospinal fluid cytology and biopsy, followed by high-dose methotrexate chemotherapy and radiotherapy. Pena et al further reported a secondary lymphoma mimicking a “dumbbell-shaped” schwannoma.^[9]^ These findings underscore the imperative to include lymphoma in the differential diagnosis of Meckel cave lesions. Cerebrospinal fluid cytological analysis and targeted biopsy are essential to exclude lymphoma, enabling precision therapy while avoiding non-indicated surgeries.^[10,11]^

CNSL classically demonstrates isodense to hyperdense attenuation on computed tomography, hypointense to isointense signal on T1-weighted imaging, and hyperintense signal on T2-weighted imaging. These lesions typically exhibit mild perilesional edema and mass effect, with calcification, hemorrhage, and necrosis being uncommon.^[12]^ Characteristic restricted diffusion on diffusion-weighted imaging (DWI), presenting as hyperintensity, is attributable to densely packed tumor cells with elevated nuclear-to-cytoplasmic ratios that severely restrict water molecule diffusion within the hypercellular tumor microenvironment.

In this case, DWI demonstrates regional signal discordance (Fig. 1). The solidly enhancing component exhibits mild hyperintensity (suggesting restricted diffusion), corresponding to a reduced apparent diffusion coefficient (ADC) value of 0.721 × 10⁻³ mm²/s. This measured value exceeds the typical ADC range for classic CNSL (0.60 to 0.70 × 10⁻³ mm²/s).^[13]^ Conversely, the necrotic region displays DWI hypointensity with elevated ADC (1.05 × 10⁻³ mm²/s) due to enhanced free-water diffusion and predominant T2 shine-through effect. This quantitative divergence precisely correlates with pathological findings: The attenuated diffusion restriction in solid regions likely originates from reduced cellular attenuation in the GCB-subtype lymphoma compared to non-GCB variants, while the elevated ADC in necrosis (1.05 × 10⁻³ mm²/s) corresponds to liquefactive necrosis foci.^[14]^

Radiologically, distinguishing this lymphoma from trigeminal schwannoma was challenging. The mass, centered in the lateral wall of the cavernous sinus, extended bidirectionally: posteriorly through Meckel cave into the posterior fossa (cerebellopontine angle) and anteriorly via the foramen ovale into the infratemporal fossa (between the tensor veli palatini and lateral pterygoid muscles). This formed a classic “dumbbell-shaped” transcompartmental lesion exhibiting perineural spread along the trigeminal nerve – a feature historically considered pathognomonic for schwannoma.^[15]^ This case refutes the traditional dogma that “perineural growth indicates schwannoma,” demonstrating lymphoma’s capacity for identical growth patterns and underscoring the need for updated diagnostic strategies in this region. Key radiological discriminators include schwannomas typically showing marked T2 hyperintensity, frequent cystic/necrotic changes, and mild-to-moderate homogeneous enhancement, whereas this lymphoma demonstrated T1/T2 isointensity (similar to cortex) and markedly heterogeneous enhancement – critical differentiating features. Furthermore, while the MRI signal characteristics of this lesion resemble those of meningioma, key differentiating features argue against this diagnosis. Notably, the lesion demonstrates no significant “dural tail sign,” absence of bony hyperostosis, and no encasement of the internal carotid artery – thereby effectively excluding meningioma in the context of this lesion.^[16]^

Due to preoperative misdiagnosis as schwannoma, the patient underwent resection – a deviation from CNSL management principles that may compromise prognosis. Surgery delays chemotherapy and risks complications. While whole-brain radiotherapy (WBRT) historically served as the therapeutic mainstay with > 80% initial response rates, most patients experienced rapid recurrence and median overall survival remained limited to 12 to 17 months; consequently, WBRT no longer constitutes first-line therapy.^[17]^ High-dose methotrexate (HD-MTX)-based combined immunochemotherapy now represents the established first-line regimen for CNSL patients.^[18,19]^ However, preoperative decision-making relied on cardinal imaging features highly suggestive of schwannoma – specifically, transcompartmental “dumbbell” morphology, perineural extension along the trigeminal course, and foraminal enlargement at the ovale. Coupled with the extreme rarity of lymphoma at this site, clinicians omitted lymphoma from differential considerations. To decompress the mass effect and alleviate neuropathic facial pain, the patient ultimately underwent surgical intervention without prior cerebrospinal fluid cytology or diagnostic biopsy. Consequently, accurate recognition of such imaging patterns should heighten clinicians’ vigilance for lymphoma, enabling earlier diagnosis and optimized therapeutic planning.

The focal CD5 positivity on immunohistochemistry warrants particular vigilance, as CD5 typically serves as a marker for chronic lymphocytic leukemia and mantle cell lymphoma, though occasionally expressed in DLBCL Compared to CD5-negative DLBCL patients, de novo CD5-positive DLBCL demonstrates inferior treatment response and frequent relapses.^[20]^

## 4. Limitations

This single-case report has inherent limitations. The extraordinary rarity of Meckel cave lymphoma requires confirmation of our imaging findings in larger studies. In addition, the lack of preoperative cerebrospinal fluid flow cytometry and whole-body PET-CT led to incomplete disease staging. Thus, the generalizability of the diagnostic and management insights presented here remains limited. Although this case was selected for its illustrative value in highlighting diagnostic pitfalls, it reflects only 1 presentation of a pathologically heterogeneous disease.

## 5. Conclusion

In the differential diagnosis of Meckel cave lesions, lymphoma must be considered, particularly when a lesion exhibits “dumbbell-shaped” growth (mimicking trigeminal schwannoma) with symptom duration < 6 months. If the lesion involves the trigeminal nerve and causes additional cranial neuropathies unexplained by imaging, this strongly suggests malignant dissemination. Based on clinical and radiological features, lesion biopsy should be prioritized when lymphoma is suspected preoperatively. Biopsy is paramount when lymphoma is suspected. Confirmed cases require combined radiotherapy and chemotherapy. Despite the clear clinical implications outlined, these conclusions are primarily derived from the analysis of a single, albeit highly informative, case and warrant further investigation through larger multi-center studies.

## Author contributions

**Conceptualization:** Qiang Li.

**Data curation:** Xiaowen Liu.

**Investigation:** Xiaowen Liu.

**Methodology:** Chengxin Yan.

**Resources:** Qinghua Zhang.

**Writing – original draft:** Tong Zhang.

**Writing – review & editing:** Chengxin Yan.
